# The Characteristics of Ocular Findings and the Presence of SARS-CoV-2 in the Tears of Coronavirus Disease 2019 Patients

**DOI:** 10.7759/cureus.44589

**Published:** 2023-09-02

**Authors:** Kanishk Singh, Rajesh Pattebahadur, Vishal Shete, Puja Bang, Meena Mishra, Neeta Gade

**Affiliations:** 1 Ophthalmology, All India Institute of Medical Sciences, Nagpur, IND; 2 Microbiology, All India Institute of Medical Sciences, Nagpur, IND

**Keywords:** rt-pcr, conjunctival swab, tear film, ocular manifestation, covid-19

## Abstract

Purpose

The purpose of the study is to observe the characteristics of ocular manifestations in coronavirus disease 2019 (COVID-19) patients and to analyze the presence of severe acute respiratory syndrome coronavirus 2 ribonucleic acid (SARS-CoV-2 RNA) in the tears of patients with moderate-to-severe COVID-19.

Material and methods

We conducted this prospective cross-sectional study from February to June 2021 at the All India Institute of Medical Sciences, one of the tertiary eye care centers in Nagpur, India. The study included confirmed COVID-19 patients based on real-time reverse transcription-polymerase chain reaction (RT-PCR) nasopharyngeal swabs, whether or not the patients exhibited ocular symptoms. We recorded detailed information regarding the patients’ history, including demographic profile, ocular symptoms, systemic symptoms, and radiologic findings. We collected ocular samples within 48 hours of collecting naso-oropharyngeal samples from the patients’ eyes. We used conjunctival swabs to obtain tear samples, which we then placed in viral transport media (VTM) for cold chain transportation to the microbiology department. We performed RT-PCR on the tear samples to detect the presence of the SARS-CoV-2 virus.

Result

We included 40 patients in the study, with 26 (65%) classified as having moderate COVID-19, six (15%) classified as having severe COVID-19, and the remaining having mild COVID-19. Out of the 40 patients, five (12%) tested positive for SARS-CoV-2 in the tear sample using RT-PCR, seven (17%) exhibited ocular signs and symptoms, and only one tested positive for SARS-CoV-2 in their tears. The ocular manifestations observed in COVID-19 patients included dry eye, conjunctivitis (including conjunctival hyperemia and epiphora), and lid edema. Notably, we detected a positive COVID-19 tear sample in patients both with and without ocular symptoms.

Conclusion

Limited reports have focused on ocular involvement in patients with COVID-19. However, our study demonstrates the detection of SARS-CoV-2 in conjunctival swabs from confirmed COVID-19 patients, albeit with a lower positivity rate. Despite the low prevalence of the virus found in tears, there is a potential risk of transmission through ocular routes. It is noteworthy that we observed a COVID-19-positive tear sample in patients with and without ocular symptoms. Therefore, it is important to consider the possibility of ocular transmission even in the absence of ocular manifestations. Medical personnel should take careful precautions during ocular examinations of patients diagnosed with COVID-19 to minimize the risk of transmission.

## Introduction

The coronavirus disease 2019 (COVID-19) pandemic, which originated in Wuhan, China, in December 2019, has rapidly spread worldwide. This highly contagious disease can lead to severe respiratory distress syndrome and even death [1,2]. The causative agent of this illness is a new coronavirus known as severe acute respiratory syndrome coronavirus 2 (SARS-CoV-2) [3]. Common symptoms include fever, cough, cold, and respiratory issues such as shortness of breath. Additionally, studies have shown that COVID-19 can also affect the eyes, causing conjunctivitis with symptoms ranging from redness to excessive tearing, to swelling [2,4]. Despite COVID-19’s significance, the ocular manifestations of the disease have not received sufficient attention in international guidelines. Some researchers have suggested the presence of the virus in tears and conjunctival secretions, raising concerns about possible transmission through these routes [5,6]. However, further investigation is necessary to determine the viral loads in ocular tissues. In this study, we aim to shed light on potential ocular manifestations of SARS-CoV-2 infection and to offer a starting point for further research on ocular involvement and its implications in humans.

## Materials and methods

**Figure 1:**
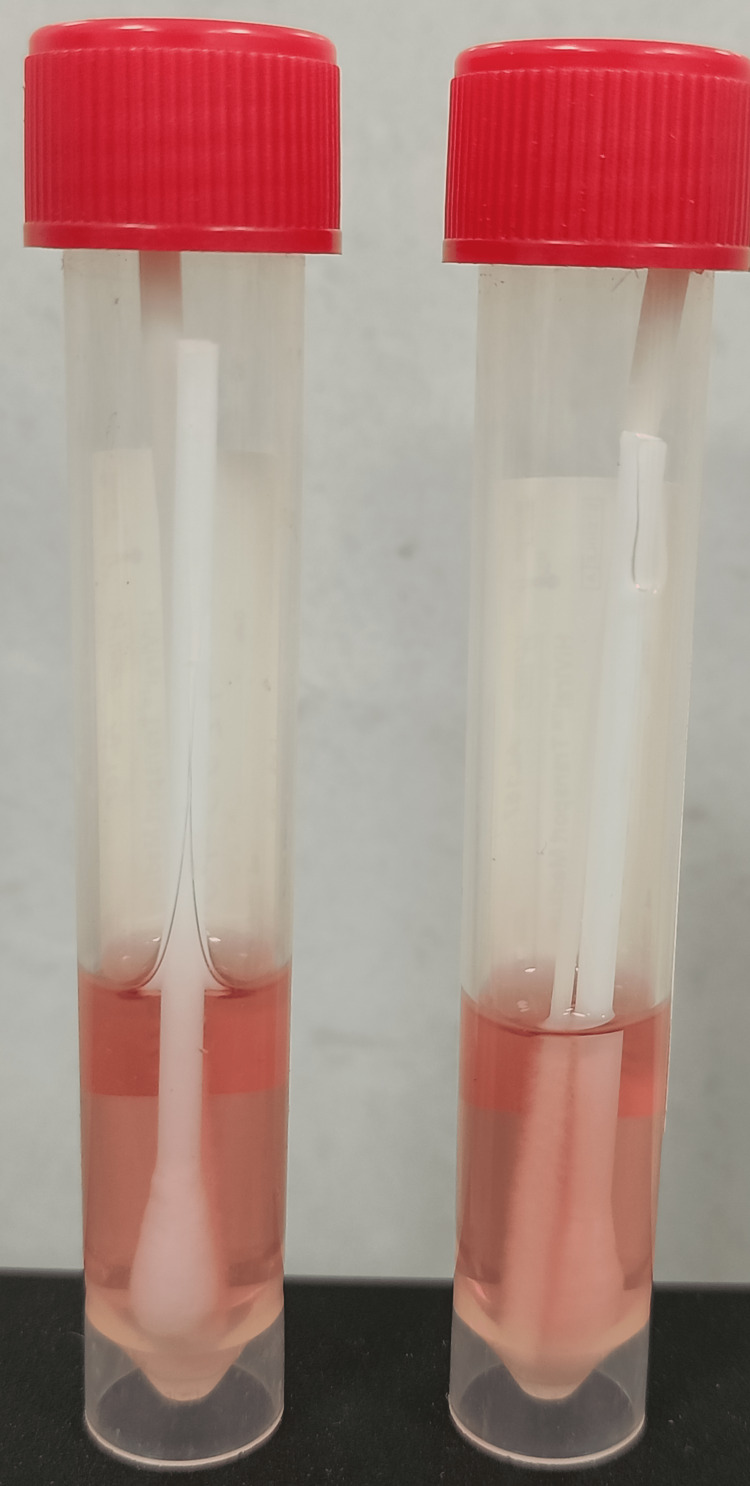
Viral transport media (VTM) with conjunctival swab

We conducted this cross-sectional study at the All India Institute of Medical Sciences, a prominent tertiary eye care center in Nagpur, India. We conducted the study between February and June 2021, following the necessary ethical clearance. All the participants were classified into mild, moderate, and severe cases of COVID-19 as per clinical management protocol: COVID-19, Ministry of Health and Family Welfare guidelines, Government of India. Patients were confirmed through a positive nasopharyngeal real-time reverse transcription-polymerase chain reaction (RT-PCR) assay performed at least 24 hours prior to admission. We included only patients who exhibited ocular symptoms or were confirmed cases of COVID-19 through nasopharyngeal swabs. We collected detailed information such as demographic profile, history of exposure, ocular and systemic symptoms, and radiologic findings; any significant past or medical history was also noted. The exclusion criteria consisted of asymptomatic individuals, uncooperative patients, and those with critical COVID-19. We also excluded patients with preexisting chronic eye conditions (e.g., uveitis, glaucoma, and dry eye), patients with contact lenses, and patients who recently used allergic or antibiotic eye drops. We included cooperative individuals aged 15 years and above with a positive RT-PCR report for COVID-19 (nasopharyngeal swab). We obtained a comprehensive ocular history and conducted a thorough ocular examination on each patient. To collect ocular samples, the ophthalmologist wore personal protective equipment and obtained samples from both eyes within 48 hours of collecting naso-oropharyngeal samples. The ophthalmologist performed the procedure without using any topical anesthetic and used conjunctival swabs to collect tear samples by retracting the lower eyelid and gently sweeping a sterile swab across the inferior fornix of each eye for a duration of 10 seconds. We then placed the conjunctival swabs from both eyes in viral transport media (VTM) and processed the swabs for nucleic acid extraction using a viral nucleic acid extraction kit (Figure 1).

**Figure 2:**
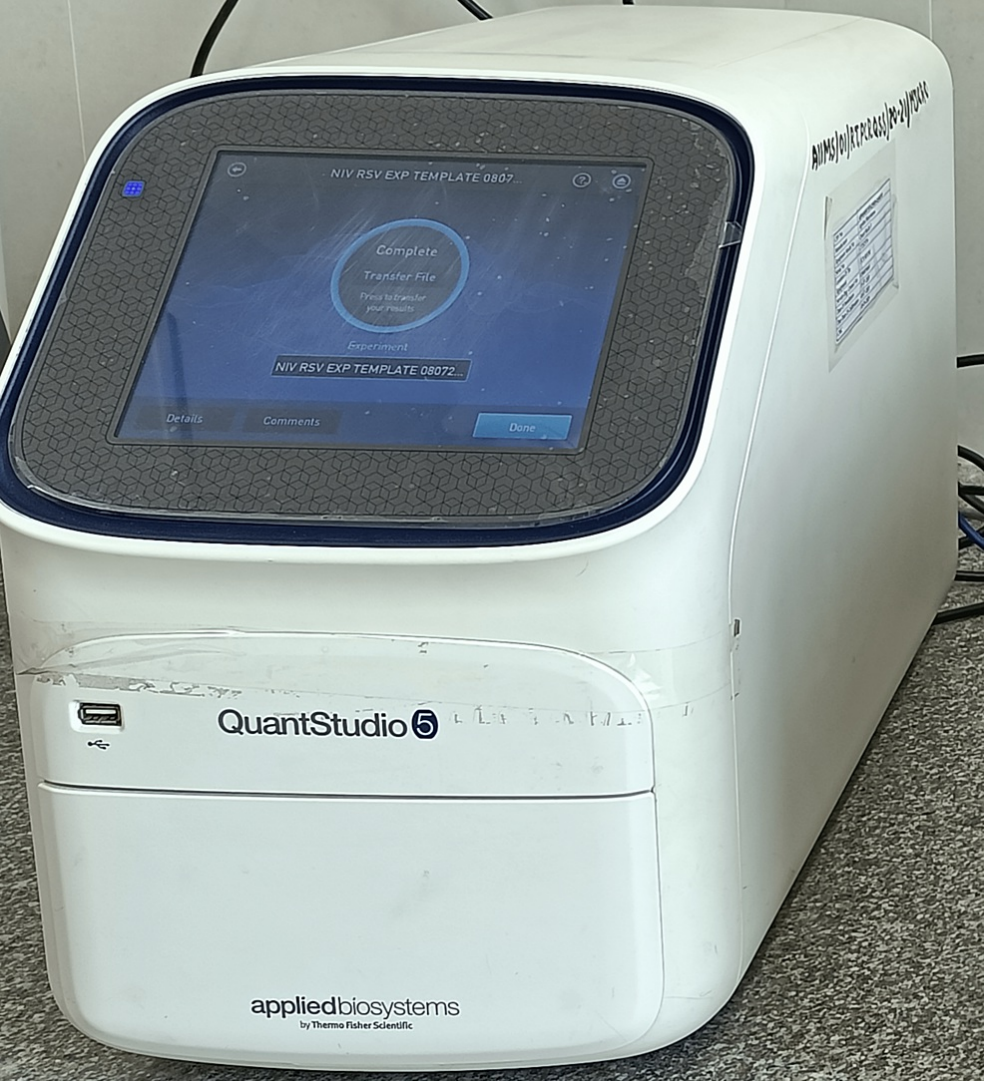
Reverse transcription-polymerase chain reaction (RT-PCR) machine

We analyzed the extracted ribonucleic acid (RNA) using the Meril COVID-19 One-Step RT-PCR Kit (Vapi, India) and a double-gene detection system (*N* gene and *ORF1ab* gene). Following the kit protocol, we considered a sample to be positive if the cycle threshold (CT) value was ≤36 and negative if the CT value was >36 (Figure 2).

## Results

**Figure 3:**
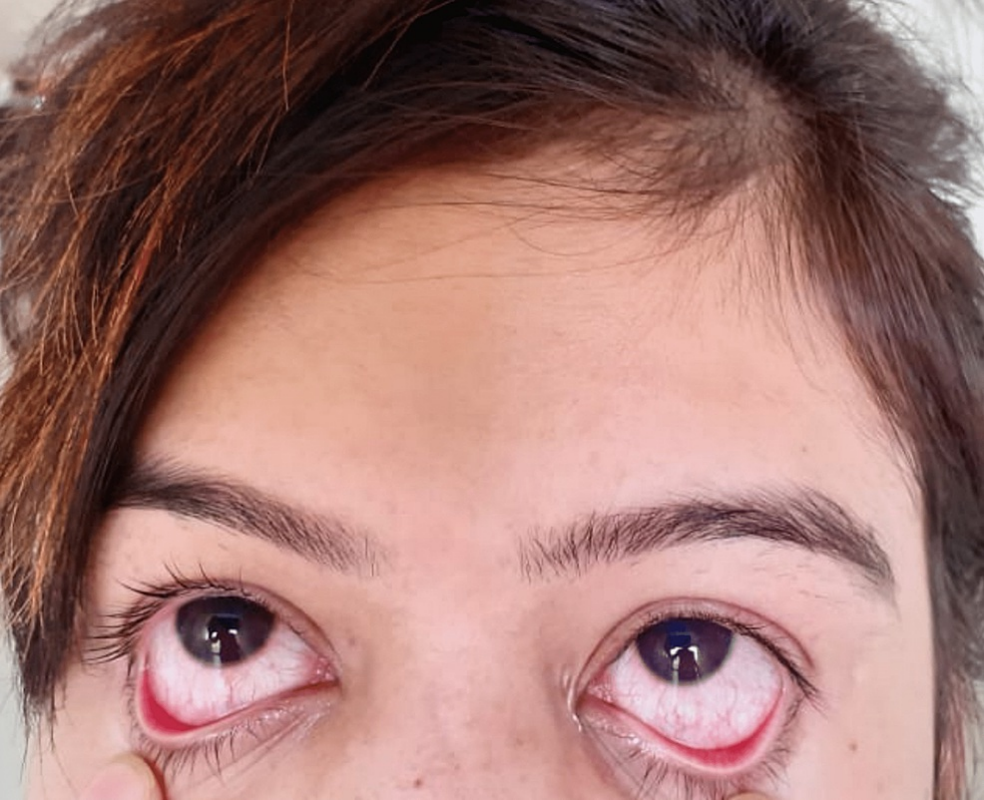
Conjunctivitis

We enrolled 40 patients, including 23 males and 17 females, in the study. Their ages ranged from 22 to 77 years. Twenty-six of the patients (65%) had moderate COVID-19, six (15%) had severe COVID-19, and the remainder had mild COVID-19. Five patients (12%) tested positive for RT-PCR in their tear samples. The ocular manifestations observed in these patients were consistent with dry eye and conjunctivitis, which included conjunctival hyperemia, epiphora, and one case of lid edema. Seven patients (17%) exhibited ocular signs and symptoms, but only one tested positive for RT-PCR in their tears. Among these seven patients, two had conjunctivitis, and five experienced dryness and epiphora, with one also presenting with lid edema. Of these seven patients, one had mild COVID-19, four had moderate COVID-19, and two had severe COVID-19 (Figure 3 and Table 1).

**Table 1:** Demography, oxygen saturation, clinical symptoms, nasopharyngeal swab, and conjunctival swab reports of COVID-19 patients SOB, shortness of breath; COVID-19, coronavirus disease 2019

Serial number	Age (year)	Sex	Saturation	Clinical symptom	Ocular symptoms	Nasopharyngeal swab	Conjunctival swab
1	35	Female	98%	Fever, cold, and cough	None	Positive	Negative
2	65	Male	91%	Fever, cough, and SOB	None	Positive	Negative
3	59	Female	90%	Fever, cold, and cough	None	Positive	Negative
4	63	Male	95%	Fever, cold, and cough	None	Positive	Positive
5	77	Female	65%	Fever, cough, and SOB	Watering	Positive	Negative
6	57	Female	80%	Fever, cough, and SOB	None	Positive	Negative
7	43	Female	90%	Cough and sore throat	Conjunctivitis, redness, and watering	Positive	Negative
8	60	Male	70%	Fever, diarrhea, and SOB	None	Positive	Negative
9	38	Male	88%	Fever, cough, sore throat, and SOB	None	Positive	Negative
10	76	Female	98%	Fever	None	Positive	Negative
11	53	Male	96%	Cold and cough	None	Positive	Negative
12	42	Male	94%	Fever	None	Positive	Negative
13	59	Male	85%	Fever, diarrhea, and SOB	None	Positive	Negative
14	65	Female	92%	Fever	None	Positive	Positive
15	74	Female	94%	Fever and cough	None	Positive	Negative
16	65	Female	82%	Fever, cold, and SOB	None	Positive	Negative
17	70	Male	90%	Fever and cough	Dryness/watering	Positive	Negative
18	65	Female	68%	Headache, fever, cough, and SOB	None	Positive	Positive
19	61	Female	88%	Fever, cough, and SOB	Watering and itching	Positive	Positive
20	22	Male	94%	Fever, headache, and SOB	None	Positive	Negative
21	31	Male	88%	Fever and SOB	None	Positive	Negative
22	35	Male	98%	Cough and sore throat	None	Positive	Negative
23	64	Male	70%	Fever, cold, cough, and SOB	Watering/itching	Positive	Negative
24	61	Male	87%	Fever, cold, cough, and SOB	None	Positive	Negative
25	67	Male	93%	Cold, cough, and SOB	None	Positive	Negative
26	65	Male	90%	Fever, cold, and cough	None	Positive	Negative
27	65	Male	88%	Fever	None	Positive	Negative
28	38	Male	92%	Headache, cold, cough, and SOB	None	Positive	Negative
29	24	Female	98%	Fever	Conjunctivitis, redness, itching, and watering	Positive	Negative
30	72	Male	74%	Fever, cough, and SOB	None	Positive	Negative
31	44	Female	87%	Fever, headache, and SOB	None	Positive	Positive
32	42	Male	92%	Cold, cough, and fever	None	Positive	Negative
33	58	Female	95%	Fever	None	Positive	Negative
34	59	Male	85%	Fever, diarrhea, and SOB	None	Positive	Negative
35	32	Female	88%	Fever and cold	None	Positive	Negative
36	43	Male	92%	Fever, cold, and cough	None	Positive	Negative
37	44	Female	74%	Headache, fever, cough, and SOB	None	Positive	Negative
38	59	Male	88%	Fever	Itching, watering, and lid edema	Positive	Negative
39	43	Female	97%	Headache, cold, and cough	None	Positive	Negative
40	64	Male	90%	Cold, cough, and fever	None	Positive	Negative

## Discussion

Few researchers have cited evidence of the presence of coronavirus in conjunctival secretions and tear samples, but its transmission via ocular secretions is still subject to debate. Five patients (12%) in this study tested positive for RT-PCR in their tears. The presence of SARS-CoV-2 in tears could relate to the infection of the lacrimal gland, the passage of the virus from the respiratory tract to the nasolacrimal duct, or direct inoculation [7]. However, the low level of viral detection in tear samples may be influenced by various factors, including the maximum replication time of the virus, the timing of sample collection, and the patient’s presentation time at the hospital [8,9]. In this study, out of the seven patients (17%) who exhibited ocular manifestations such as conjunctivitis, conjunctival hyperemia, epiphora, and dry eye symptoms, only one patient tested positive for RT-PCR in their tears. Several factors may explain the limited presence of the virus in tears. First, the lacrimal duct constantly renews and drains tears, resulting in a relatively low volume. Second, ocular secretions possess a robust local immune system comprising lactoferrin, immunoglobulins, sialic acid, and other components that provide protection against microorganisms, including coronaviruses. Third, researchers have postulated that the angiotensin-converting enzyme 2 (ACE2) receptors, to which the virus binds to enter host cells, are present in the eye in smaller amounts compared to other tissues, and their binding capability to the virus is relatively poor, being at least 50% less. Other factors, such as the stage of the disease, technical errors and difficulties, and the handling and processing of samples, may contribute to the lower levels of virus detected in tears [10-13].

Rai et al. [14] and Arora et al. [15] found higher rates of positive results in tear samples reported at 23.33% and 24%, respectively. Conversely, Rodríguez-Ares et al. [16] and Karimi et al. [4] found lower rates at 7.1% and 7%, respectively, which closely align with our findings. The detection of viral RNA in tears has varied across studies, ranging from 0% to 7%. Kumar et al. (2.23%) [8] and Wu et al. (5.2%) [3] suggested minimal viral shedding in ocular secretions. We observed a slightly higher positivity rate in the RT-PCR assay for SARS-CoV-2 in tear samples, perhaps because we conducted sampling within 48-72 hours of admission when viral load tends to be high. Additionally, the sampling technique involved swabs from both eyes, ensuring prolonged contact with the tear film. Racial variations in different regions and the prevalence of different virus subtypes may also contribute to the variation in positivity rates. The specific incidence of ocular manifestations associated with COVID-19 remains unclear; however, some epidemiological data indicate that the incidence of conjunctivitis in COVID-19 patients ranges from 0.82% to 4.76% [2,5,17]. According to Chen et al. [2], the common ocular symptoms of COVID-19 include conjunctival congestion (4.68%), dry eye (20.97%), blurred vision (12.73%), and foreign body sensation (11.80%), which align with our findings. In another study, Wu et al. [3] found that 12 out of 38 patients with ocular manifestations exhibited symptoms consistent with conjunctivitis, such as conjunctival hyperemia, chemosis, epiphora, and dry eye. In our study, out of the seven patients with ocular manifestations, two patients had conjunctivitis, and five experienced dryness and epiphora, with one patient also presenting with lid edema. This finding suggests that viral shedding in tears is not always indicative of ocular involvement, as some studies have suggested [8]. It is important to note that our study has certain limitations. These limitations include a small sample size and a single-time sampling approach, which may affect the homogeneity of our results. Because of logistical challenges, we did not perform detailed ocular examinations, such as a slit lamp examination and other intraocular assessments, to investigate potential intraocular associations.

## Conclusions

The study’s findings indicated that among a total of 40 patients, 12% (n=5) tested positive for SARS-CoV-2 in RT-PCR tear samples. Among these five positive cases, only 20% (n=1) displayed ocular manifestations, while the remaining 80% (n=4) did not exhibit any signs of ocular symptoms. Additionally, the study revealed that out of the seven patients (17%) who showed ocular symptoms such as conjunctival hyperemia, epiphora, watering, and itching, only 14% (n=1) tested positive for SARS-CoV-2 in RT-PCR tear samples. The remaining six patients tested negative for SARS-CoV-2 in tear samples.

It is worth noting that the viral load in conjunctival samples is generally lower when compared to nasopharyngeal secretions. Despite this disparity, concerns persist regarding the potential transmission of the disease through tear samples, even when obvious ocular signs and symptoms are absent. Thus, it is emphasized that relying solely on tear samples for diagnosing COVID-19 should be avoided, and they cannot be considered a substitute for other established diagnostic methods.
